# Comparison between Double J (DJ) Ureteral Stenting and Percutaneous Nephrostomy (PCN) in Obstructive Uropathy

**DOI:** 10.12669/pjms.293.3563

**Published:** 2013

**Authors:** Iftikhar Ahmad, Mudassar Saeed Pansota, Muhammad Tariq, Muhammad Shahzad Saleem, Shafqat Ali Tabassum, Akbar Hussain

**Affiliations:** 1 Dr. Iftikhar Ahmad, FCPS, Assistant Professor, Department of Urology and Renal Transplantation, Bahawal Victoria Hospital/Quaid-e-Azam Medical College, Bahawalpur, Pakistan.; 2 Dr. Mudassar Saeed Pansota Post Graduate Resident, Department of Urology and Renal Transplantation, Bahawal Victoria Hospital/Quaid-e-Azam Medical College, Bahawalpur, Pakistan.; 3 Dr. Muhammad Tariq, FCPS, Medical Officer, Department of Urology and Renal Transplantation, Bahawal Victoria Hospital/Quaid-e-Azam Medical College, Bahawalpur, Pakistan.; 4 Dr. Muhammad Shahzad Saleem, FCPS, Medical Officer, Department of Urology and Renal Transplantation, Bahawal Victoria Hospital/Quaid-e-Azam Medical College, Bahawalpur, Pakistan.; 5 Prof. Dr. Shafqat Ali Tabassum, FCPS, Head of Department of Urology and Renal Transplantation,; 6 Dr. Akbar Hussain, FCPS, Medical Officer, Department of Urology and Renal Transplantation, Bahawal Victoria Hospital/Quaid-e-Azam Medical College, Bahawalpur, Pakistan.

**Keywords:** Double J stenting, Percutaneous nephrostomy, Obstructive uropathy, Complications rate

## Abstract

***Objective:*** To compare the complications rate of percutaneous nephrostomy and double J ureteral stenting in the management of obstructive uropathy.

***Methodology: ***Total number of 300 patients of age 20-80 years who underwent JJ stenting or percutaneous nephrostomy for obstructive uropathy were included in this study. Patients were divided in two groups i.e. A & B. In group A, 100 patients who underwent double J ureteral stenting while in group B, 200 patients who underwent percutaneous nephrostomy tube insertion were included. The stent was inserted retrograde by using cystoscope, under mild sedation or local anesthesia. While the percutaneous nephrostomy was done under ultrasound guidance by using local anesthetic agent. Complications were noted in immediate post-operative period and on follow up.

***Results:*** Majority of the patients were between 36 to 50 years of age with male to female ratio was 2.6:1. The most common cause of obstructive uropathy was stone disease i.e. renal, ureteric or both. Post DJ stent, complications like painful trigon irritation, septicemia, haematuria and stent encrustation were seen in 12.0%, 7.0%, 10.0% and 5.0% patients respectively. On the other hand, post-PCN septicemia, bleeding and tube dislodgment or blockage was seen in 3.5%, 4.5% and 4.5% respectively. In this study, overall success rate for double J stenting was up to 83.0% and for percutaneous nephrostomy (PCN) was 92.0% (p<0.0001).

***Conclusion: ***Percutaneous nephrostomy is a safe and better method of temporary urinary diversion than double J stenting for management of obstructive uropathy with lower incidence of complications.

## INTRODUCTION

Obstructive uropathy is the structural impedance to the flow of urine and can occur at any level from uretheral meatus to the calyceal infundibula. It refers to the pathophysiolocial effects secondary to this obstruction leading to renal dysfunction.^1^ The obstruction may be due to intraluminal, intramural and extramural causes. In young and middle age patients' renal calculi are the main etiological factors of obstruction.^2^ In female, Gynaecological tract obstruction and obstetrical trauma while in old people, malignancy contributes to upper obstructive uropathy.^2^^,^^3^

It is a potentially life threatening condition and if the obstruction is present bilaterally, then immediate measures are required to decompress the kidney, otherwise the patient’s clinical conditions will deteriorate at a fast pace^4^ through uremia, water-electrolyte abnormalities and urinary infections with a consequent reduction of alertness and subsequent death.^3^^,^^5^ Urinary diversion is one of the ways to manage ureteral obstructions and is commonly performed in our daily practice when the underlying pathology of ureteral obstruction cannot be eliminated in a short period. The various methods of urinary diversions are retrograde double J ureteral stenting, percutaneous nephrostomy and open drainage of kidney.^2^^,^^6^

Clear guidelines regarding optimal urinary diversions have not been established. Most authors agreed that decisions should be individualized.^7^ Currently, retrograde double-J ureteral stenting and ultrasound guided percutaneous nephrostomy tube insertion are the most widely used techniques for relieving obstruction of the urinary tract.^6^^,^^7^ Both are associated with variable technical success, complication rates, availability and quality of life issues. Retrograde implantation of ureteral stents is associated with septicemia, irritative bladder symptoms, forgotten stents and high failure rate which ultimately require percutaneous nephrostomy tube insertion to drain the affected kidney.^8^ On the other hand, Percutaneous nephrostomy is associated with complications like bleeding, septicemia, tube blockage and accidently tube dislodgement. ^2^^,^^5^^,^^7^^,^^10^ Moreover, PCN also requires an extra care of external urine-collecting bag.

This study was conducted to compare the complications rate of ultrasound guided percutaneous nephrostomy (PCN) and double J (DJ) ureteral stenting in the management of obstructive uropathy.

## METHODOLOGY

This study was conducted at the Department of Urology & Renal Transplantation, Bahawal Victoria Hospital/Quaid-e-Azam Medical College, Bahawalpur from January 2010 to December 2011. After approval from ethical review committee, total number of 300 patients of obstructive uropathy who underwent double J stenting or percutaneous nephrostomy were included in this study. Informed, written consent was taken from each patient before the procedure after explaining all merits and demerits of the procedure. Patients with severe coagulopathies and uremia due to bladder outflow obstruction were excluded from the study. Patients were divided in two groups i.e. A & B by using random number tables. In group A, 100 patients who underwent double J ureteral stenting while in group B, 200 patients who underwent percutaneous nephrostomy tube insertion were included. Detailed history and physical examination of every patient was done. The investigations done before the procedure were complete blood count, urine complete examination, screening for Hepatitis B & C and serum urea and creatinine. Abdominal ultrasonography was done in every patient to see the degree of hydronephrosis and the side affected.

***Procedural Detail: ***In group A, the double J ureteral stent was inserted retrograde by using cystoscope, under mild sedation or local anesthesia by instilling 2% xylocain gel per urethra. Patients who were not infected received a single prophylactic dose of intravenous antibiotics two hours before stent insertion. Infected patients had the DJ stenting, covered by specific antimicrobial therapy according to urine and/or blood culture. This treatment continued until there was no fever and any evidence of infection disappeared. A Foley’s catheter was left in the bladder for 48 hours in all patients for Intake Output record and any hematuria. In each case the type of stent inserted was that of 5 or 6 F, with side-holes and remain in place for either 6 weeks or longer according to the pathology necessitating stenting.

In group B, percutaneous nephrostomy (PCN) tube was inserted under ultrasound guidance by using 5-10ml of 1% lignocaine subcutaneously at the puncture site. All the patients were given non-nephrotoxic antibiotics pre-operatively. The patients were placed on the ultrasound table in prone position and a pillow placed under the abdomen on the affected side to support the kidney. Then the initial puncture site was chosen, cleaned and draped. Local anesthesia was injected and a stab incision was given at the puncture site. The 18-gauge Chiba needle was inserted at the renal angle or at the posterior axillary line under ultrasound guidance into dilated pelvicalyceal system. Urine or pus drained out spontaneously or was sucked with a disposable syringe and sample was sent to the laboratory for culture and sensitively. Then soft end of floppy J guide wire was passed through the needle and needle was removed. The tract was dilated with Teflon facial dilators more than the diameter of the nephrostomy tube. After tract dilation a pig tail nephrostomy tube or a feeding tube of 8 Fr was passed over the guide wire into the collecting system and secured with silk no. 1.

All patients were maintained on antibiotic prophylaxis. Complications were noted in immediate post-operative period and on follow up. All patients were scheduled to undergo removal or replacement of the stent according to the specific pathology or type of stent. Patients with complications were immediately hospitalized and managed accordingly. Minimum follow up period was 15 days and maximum 3 months for these particular patients. The collected information was analyzed by computer software SPSS version 16. Chi Square was applied to compare the complications rate. P value ≤ 0.05 was considered as significant.

## RESULTS

Age range was from 20 to 80 years with mean age of 43±9.65 years in group A patients and 40 ± 10.35 year in group B patients (p<0.0001). Age of the patients at presentation is shown in Table-I. Out of these 300 patients, 72.67% were male and 27.33% female with male to female ratio of 2.6:1. 

The most common cause of obstructive uropathy was stone disease i.e. renal, ureteric or both and 75.0% patients in group A and 65.0% in group B, presented with it followed by other causes i.e. carcinomas, pyonephrosis and PUJ obstruction as shown in Table-II.

**Fig.1:**
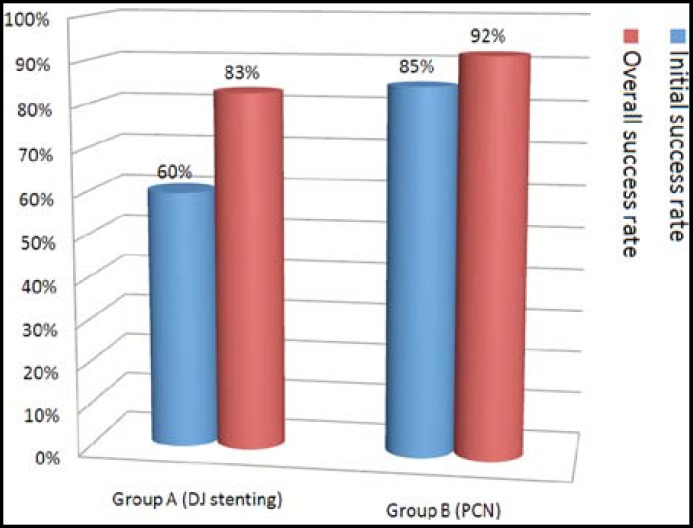
Success rate of both groups

The post-operative complications are shown in Table-III. After double J ureteral stenting (Group A), Fever and septicemia occurred in 07 patients. It was managed conservatively by injectable antibiotics and anti-pyretic in 05 cases except 02 cases in which stents had to be removed. Haematuria was seen in 10 patients, which was settled within 24 hours in 08 patients by giving I.V. fluids, while 02 patients required haemostatic agents and blood transfusion. Painful trigon irritation was common and distressing in 12 patients which were settled by anti-cholinergics in 10 patients, while in 02 patients it resulted in early DJ Stent removal. Ureteral perforation occurred in only 01 patient in which immediately DJS was removed. Upward stent migration was seen in 02 patients in whom endoscopic removal was done. Encrustation occurred in five patients who were lost to follow up. Three cases were managed by ESWL breaking up the encrustation and later on removal of the stent while in 02 patients open surgery was done due to stone formation on the stent. Procedural failure occurred in 03 patients in which PCN had to be done later on. Initial success rate was 60.0% but 23 patients with bleeding, septicemia and trigon irritation were managed conservatively giving overall success rate of 83.0% (Fig.1). On the other hand, in percutaneous nephrostomy (Group B), bleeding and septicemia occurred in 09 and 07 patients respectively. They were managed conservatively by giving I.V, fluids in 07 cases, while hemostatics and blood transfusion was required in 02 cases of bleeding and by giving injectable antibiotics in cases of septicemia. PCN dislodgement or blockage occurred in 09 patients which were managed by re-inserting nephrostomy tube. PCN procedural failure occurred in 05 patients which were then subjected to renal replacement therapy. Initial success rate was 85.0% but patients with bleeding and septicemia were managed conservatively so overall success rate was 92.0% (Fig.1) (p<0.0001). Out of these 300 patients, 77.0% patients had undergone definitive procedure after settlement of their general condition and renal parameters. While 8.0% patients passed their stone spontaneously. The remaining patients were declared as end stage renal disease and subjected to renal replacement therapy.

**Table-I:** %age of patients according to age group (n=300).

*Age (years)*	*Group A (n=100)*		*Group B (n=200)*	
	*No. of patients*	*%age*	*No. of patients*	*%age*
20-35	21	21.0	50	25.0
36-50	40	40.0	78	39.0
51-65	24	24.0	46	23.0
66-80	15	15.0	26	13.0
Total	100	100.0	200	100.0

**Table-II:** Causes of Obstructive Uropathy (n=300)

*Causes*	*Group A (n=100)*		*Group B (n=200)*	
	*No. of patients*	*%age*	*No. of patients*	*%age*
*Stone disease* Renal Ureteric Renal + Ureteric	75 40 25 10	75.0 40.0 25.0 10.0	130 58 38 34	65.0 29.0 19.0 17.0
*Carcinomas* Urinary Bladder Prostate Cervix Others	20 03 02 05 10	20.0 3.0 2.0 5.010.0	36 16 08 04 08	18.0 8.0 4.0 2.0 4.0
Pyonephrosis	03	3.0	22	11.0
PUJ Obstruction	02	2.0	12	6.0

**Table-III:** Complications of both groups

*Complications*	*Group A (n=100)*		*Group B (n=200)*	
	*No. of patients*	*%age*	*No. of patients*	*%age*
Procedural failure	03	3.0	05	2.5
Fever & Septicemia	07	7.0	07	3.5
Bleeding/Hematuria	10	10.0	09	4.5
Painful Trigone Irritation	12	12.0	--	--
PCN dislodgement or blockage	--	--	09	4.5
Ureteral Perforation	01	1.0	--	--
Stent Migration	02	2.0	--	--
Injury to adjacent organs	--	--	00	0.0
Stent Encrustation or Stone formation	05	5.0	--	--
Total	40	40.0	30	15.0

## DISCUSSION

Three terms are used to describe a disease as a consequence of urinary tract obstruction: obstructive uropathy, obstructive nephropathy and hydronephrosis, but each in different connotation. If ureteral dilatation due to impaired flow of urine is associated with renal parenchymal damage, it is described as obstructive uropathy.^6^ It is a potentially life threatening condition and sometimes it is desirable to provide immediate temporary relief of the obstruction, until definitive treatment can be undertaken. Cystoscopy with retrograde catheterization (Double J Stenting) and percutaneous nephrostomy (PCN), are two main options for temporary urinary diversion with their own merits and demerits.^11^

In our study, the commonest cause of obstructive uropathy observed was stone disease (renal or ureteric) as was also found by Richter S et al^8^ and Naeem M et al.^2^ The male patients were 72.67% and female were 27.33% with ratio of 2.6:1 in this study which is very much comparable to studies of Naeem M et al^2^, Karim R et al^11^ and Memon NA et al^12^ who had also found predominance of male patients with obstructive uropathy.

Ureteral obstruction was highly amenable to endoscopic ureteral stents in cases of benign intrinsic obstruction, but the incidence of stent failure was significantly higher in cases of extrinsic compression, as was seen with most malignant diseases. It was also observed in this study that in most cases of urinary bladder or prostate carcinoma, percutaneous nephrostomy is preferable option as retrograde stenting could not be possible due to involvement of ureteric orifices by tumour. Ku JH et al^7^, Chang HC et al^13^ and Nariculam J et al^14^ had also found percutaneous nephrostomy as a better option for temporary urinary diversion in obstructive uropathy of advanced malignancies.

Double J stenting was successfully done in 96.0% of patients in our study while Memon NA et al^12^ reported as 94.2%. Those patients in which stent could not be passed or ureteric perforation occurred, were considered as unsuccessful cases and in these cases, percutaneous nephrostomy tube insertion was done to achieve urinary diversion. On the other hand, percutaneous nephrostomy (PCN) was successfully done in 97.5%% of patients in our study while Naeem M et al^2^ and Wah TM et al^15^ had come across this rate as 96.05% and 98.0% respectively. The success rate is lower in patients with non-dilated collecting system, stag horn calculi or where patient was not cooperative. The patients in whom percutaneous nephrostomy could not be possible were then subjected to renal replacement therapy.

Complications associated with the use of ureteral stents are basically mechanical in nature and are related to stent material. The most common complication was painful trigone irritation which occurred in 12.0% patients in our study. Shao Y et al^16^ and Memon NA et al^12^ have come across this rate as 10.0% and 9.0% respectively while Arshad M et al^17^ had found higher rate of bladder irritation i.e. 27.27%. The most common complication of percutaneous nephrostomy (PCN) was bleeding, which occurred in 4.5% patients in our study. Naeem M et al^2^, Jalbani MH et al^18^ and Romero FR et al^19^ had come across this rate as 4.0%, 5.0% and 3.5% respectively which is very much comparable to our study. But Karim R et al^11^ and Olivera ST et al^6^ reported a much higher rate of bleeding i.e. 9.5% and 21.5% respectively. Post DJ stenting hematuria observed in different studies range from 2-21%.^6^^,^^8^^,^^12^ In our study it was found in 10.0% patients which was settled by giving I.V. fluids in 08 patients within 24 hours, while 02 patients required blood transfusion and hemostatic agents. 

Incidence of post DJ stenting septicaemia in our study was 8.0% while Elmalik K et al^20^ reported its incidence 5.2% and Arshad M et al^17^ 10.2%. But Richter S et al^8^ reported much higher incidence of septicaemia i.e. 19.0%. On the other hand, incidence of septicemia in group B was 3.5% while Naeem M et al^2^ and Jalbani MH et al^18^ reported its incidence as 2.0% and 7.5% respectively. DJS had to be removed in 02 patients because in them fever & septicaemia could not be settled after all conservative measures.

Post PCN blockage or dislodgment of the nephrostomy tube observed in different studies range from 04-37%^2^^,^^14^^,^^15^^,^^18^ while in our study it was found in 4.5% patients. Memon NA et al^12^ and Arshad M et al^17^ observed DJ stent encrustation in 17.5%, 2.0% and stent migration in 11.7% and 16.3% respectively. In our study, stent encrustation was seen in 5.0% and stent migration in 2.0% cases. Stent encrustation and stone formation was seen more in the patients where stent indwelling period was more than three months as was also observed by other authors.^6^^,^^17^ In our study, stents remained in place for maximum of two months despite those with encrustation who had been lost to follow up. Hence stent monitoring is essential with lot of stress should be paid on the counselling of the patients regarding stents complications and their timely removal.

So, overall success rate is upto 83.0% and 92.0% respectively which is very much comparable to many previous studies.^2^^,^^7^^,^^8^^,^^13^^,^^16^ But Memon NA et al^6^ and Damiano R et al^21^ have shown a much higher complication rate of DJ stenting i.e. 79.9% and 70.0% respectively.

## CONCLUSION

This study concludes that ultrasound guided percutaneous nephrostomy is a safe, quick and better method of temporary urinary diversion than double J stenting for management of obstructive uropathy with lower incidence of complications. Moreover, PCN is also proved to be a suitable modality for drainage of pyonephrosis and ureteric obstruction especially due to malignant disease of pelvic origin which can otherwise be highly fatal.

## Contribution of Authors

IA and MSP: Conception and Design, acquisition of data, analysis and interpretation of data, drafting and critical revision, final approval of the version to be published.

MT: Acquisition of data, drafting and final approval of the manuscript.

MSS and SAT: Conception and Design, acquisition of data, drafting, final approval of the version to be published.

AH: Conception, acquisition of data, critical revision of the manuscript.
